# Expression and Immunogenicity of SARS-CoV-2 Virus-Like Particles based on Recombinant Truncated HEV-3 ORF2 Capsid Protein

**DOI:** 10.4014/jmb.2205.05023

**Published:** 2022-09-19

**Authors:** Yong-Fei Zhou, Jiao-Jiao Nie, Chao Shi, Ke Ning, Yu-Feng Cao, Yanbo Xie, Hongyu Xiang, Qiuhong Xie

**Affiliations:** 1 National Engineering Laboratory for AIDS Vaccine, School of Life Sciences, Jilin University, Changchun, Jilin 130012, P.R. China; 2 School of Life Sciences, Jilin University, Changchun 130012, P.R. China; 3 Institute of Changbai Mountain Resource and Health, Jilin University, Fusong 134504, P.R. China; 4 Immune-Path Biotechnology (Suzhou) Co., Ltd., Suzhou 215000, P.R. China; 5 Jilin Provincial Key Laboratory of Agricultural Biotechnology, Jilin Academy of Agricultural Sciences, Changchun 130033, P.R. China

**Keywords:** SARS-CoV-2, recombinant vaccine, virus-like nanoparticles, hepatitis E virus

## Abstract

COVID-19 is an emerging disease that poses a severe threat to global public health. As such, there is an urgent demand for vaccines against SARS-CoV-2, the virus that causes COVID-19. Here, we describe a virus-like nanoparticle candidate vaccine against SARS-CoV-2 produced by an *E. coli* expression system. The fusion protein of a truncated ORF2-encoded protein of aa 439~608 (p170) from hepatitis E virus CCJD-517 and the receptor-binding domain of the spike protein from SARS-CoV-2 were expressed, purified and characterized. The antigenicity and immunogenicity of p170-RBD were evaluated in vitro and in Kunming mice. Our investigation revealed that p170-RBD self-assembled into approximately 24 nm virus-like particles, which could bind to serum from vaccinated people (*p* < 0.001) and receptors on cells. Immunization with p170-RBD induced the titer of IgG antibody vaccine increased from 14 days post-immunization and was significantly enhanced after a booster immunization at 28 dpi, ultimately reaching a peak level on 42 dpi with a titer of 4.97 log_10_. Pseudovirus neutralization tests showed that the candidate vaccine induced a strong neutralizing antibody response in mice. In this research, we demonstrated that p170-RBD possesses strong antigenicity and immunogenicity and could be a potential candidate for use in future SARS-CoV-2 vaccine development.

## Introduction

Coronaviruses are single-stranded, positive-sense RNA viruses that include severe acute respiratory syndrome coronavirus (SARS-CoV) and Middle East respiratory syndrome coronavirus (MERS-CoV), both of which can cause serious respiratory system diseases [1]. In late 2019, a novel coronavirus designated as severe acute respiratory syndrome coronavirus 2 (SARS-CoV-2) emerged and caused a worldwide outbreak of viral pneumonia [2]. Pneumonia caused by SARS-CoV-2, also known as coronavirus disease 2019 (COVID-19), is highly pathogenic and has spread rapidly around the world [3]. The SARS-CoV-2 genome contains 29,903 bases, encoding approximately 9,860 amino acids [4]. Its genome sequence is highly similar to that of other coronavirus species, with approximately 80% similarity to SARS-CoV and 50% similarity to MERS-CoV [5]. Among the proteins encoded by SARS-CoV-2, the spike protein, which is the key protein that mediates virus invasion into host cells, contains two functional domains (S1 and S2). Similar to SARS-CoV, the S1 domain contains a receptor-binding domain (RBD) and can specifically bind to receptors on target cells [6].

COVID-19, caused by infection with SARS-CoV-2, can lead to severe respiratory disease, accompanied by pneumonia and severe acute respiratory syndrome [7]. Recent studies have reported that mild symptoms of SARS-CoV-2 clinical manifestations include fever and dry cough, while severe illness is associated with chronic pneumonia and severe acute respiratory symptoms, eventually generating respiratory and cardiovascular system failure [6]. As reported by the World Health Organization (WHO), SARS-CoV-2 has infected 159 million people and caused 3.3 million deaths in 212 countries/territories worldwide as of 10 May 2021, and mortality is currently estimated at approximately 2%. Since the spread of COVID-19 has had a significant impact on the economic and political landscape, there is an urgent need for an effective strategy to end this human pandemic [8].

At present, many global entities are focusing on developing vaccines for SARS-CoV-2, including inactivated vaccines, adenovirus vector vaccines, mRNA vaccines and genetically engineered recombinant protein vaccines [9]. Vaccines developed by Moderna and Pfizer were the initial mRNA vaccines that were marketed worldwide. Clinical trials showed that BNT162b2, developed by Pfizer, was 95% effective against SARS-CoV-2 [10], while mRNA-1273, developed by Moderna, was 94.1% effective [11]. There is no doubt that the development of these two vaccines was successful, both in terms of safety and immunogenicity [12], but we cannot ignore the huge difficulties in transportation, which presents a huge challenge for mass vaccination on a global scale [13]. The vaccine developed by AstraZeneca, designated ChAdOx1, used an adenovirus vector [14]; volunteers in the ChAdOx1 group received two doses containing 5×10^10^ viral particles [15], and clinical data showed that this vaccine was 79% effective against SARS-CoV-2 [16]. However, its use has been terminated in some countries due to safety issues [17]. For the inactivated vaccine on the market developed by Sinovac, it was shown to induce the seroconversion of neutralizing antibodies to SARS-CoV-2 at a rate of 100%, as demonstrated by the clinical data [18], indicating that it is a reliable vaccine, but global vaccination is still limited due to insufficient production capacity. As a type of genetically engineered recombinant protein vaccine, virus-like particle (VLP) vaccines have been widely used in research and development [19]. Since the RBD domain of SARS-CoV-2 cannot self-assemble into virus-like particles when expressed by genetic engineering, its development as a VLP-based vaccine requires another factor that can assemble into a VLP [20]. Using the properties of ferritin protein that enable it to self-assemble into 24 polymers [21], Ma X *et al*. attached the RBD to ferritin using a SpyTag/SpyCatcher system, which allowed it to assemble into particles. Animal experiments show that these particles can induce high levels of neutralizing antibodies [22]. The VLP vaccine is a viable strategy that has shown excellent prospects against SARS-CoV-2 [23], and as there are many other VLP currently vaccines under research, this indicates that VLP vaccines hold great promise [24].

Hepatitis E virus (HEV) is a nonenveloped virus with a diameter of 27-34 nm that possesses a single-stranded, positive-sense RNA genome. HEV ORF2 encodes a capsid protein consisting of 660 amino acids. Virus-like particles (aa 112-608) are made up of three definitive domains: The S domain (aa 129-319), M domain (aa 320-455), and P domain (aa 456-606), with the P domain forming protrusions that extend outward from the viral shell. Wei *et al*. confirmed that the HEV ORF2 domain consisting of aa 459 to 606 could form VLP granules on the basis of the dimer [25]. In this study, aa 439 to 608 in HEV ORF2 (hereafter referred to as p170) and RBD of SARS-CoV-2 were fused and expressed (hereafter referred to as p170-RBD), and the fusion protein could form dimers. Transmission electron microscopy revealed that p170-RBD could self-assemble into VLP particles with a diameter of approximately 24 nm. p170-RBD possessed strong antigenicity and could stimulate mice to produce a strong humoral immunity and neutralizing antibodies, providing favorable conditions for development of a vaccine.

## Materials and Methods

### Animals and Ethics

Nonpregnant female Kunming (KM) mice were all SPF grade, 6~8 weeks old, 18-22 g, and approved by the Animal Ethics Committee of Jilin University. All experimental protocols were reviewed and approved by the Animal Care and Use Committee of Jilin University (Approval No. 2021-SY0714). Mice were divided into three groups, with 6 mice per group. All efforts were made to minimize suffering during vaccination, blood collection surgery, and sacrifice.

### Human Serum Samples

Serum samples were collected from 5 healthy participants who were immunized with a commercial inactivated vaccine developed by Sinovac Biotech in China. This study was Institutional Review Board (IRB) approved by the Second Hospital of Jilin University (Approval No. 2020121). Two doses of vaccines were given intramuscularly at 0 and 1 month. Serum samples were taken before the first vaccine dose (Day 0) and one month after the second dose (Day 60). Serum samples were isolated by centrifugation at 3000 ×*g* for 10 min and stored at -20°C. Written informed consent was obtained from each participant.

### Protein 3D Structure Prediction and Simulation

The structure of the p170-RBD protein was predicted using the Phyre2 website (http://www.sbg.bio.ic.ac.uk/phyre2/html/) [26], whose server was used for protein structure prediction, and displayed with PyMol software.

### Construction of the Plasmid Vector

The SARS-CoV-2 RBD gene (GenBank: OA993881.1) and the p170 gene (GenBank: KX981911.1) were connected with a GGGGS linker and subcloned into the pET28a (+) (Thermo Fisher, USA) expression plasmid using unique the EcoRI and XhoI restriction sites to create the pET28a-p170-RBD plasmid. The p170 gene was subcloned into the pET28a expression plasmid using unique EcoRI and XhoI sites as a negative control. The ACE2 gene (GenBank: AB046569.1) was subcloned into the pcDNA3.1 (+) (Thermo Fisher) expression plasmid using unique KpnI and XhoI sites. All operations were performed in accordance with molecular experimental procedures. The general procedure was as follows: The sequence was amplified with PCR using forward and reverse primers (Table 1). After digestion with the corresponding restriction enzymes (TransGen, China), the plasmids and PCR products were recovered by a gel recovery kit (TransGen), ligated by T4 DNA ligase (TransGen) and transformed into *E. coli* DH5α competent cells (TransGen). Positive colonies were identified by PCR and sent for sequencing (Sangon Biotech, China).

### Cell Line Construction and Screening

The constructed plasmid, designated pcDNA3.1-ACE2, was transfected into 293T cells, and the protocols were as follows: 293T cells were cultured in DMEM containing 10% fetal bovine serum (FBS). Twenty-four hours before transfection, cells were cultured in 24-well plates at a density of 1 × 10^5^ cells/well. The plasmids were transfected into 293T cells with the Lipo2000 transfection reagent (Invitrogen, USA) according to the manufacturer’s instructions. Stable transformants were selected in DMEM/10% FBS supplemented with 400 μg/ml G418 (TransGen). After the selection of cells was complete, the cells were used in subsequent experiments. The expression of ACE2 was identified by western-blot and 293T cells transfected with pcDNA3.1-ACE2 or pcDNA3.1 were analyzed by SDS–PAGE and transferred to NC membranes. Then anti-ACE2 polyclonal antibody and HRP-conjunct secondary antibody were incubated respectively and reacted by ECL.

### Expression and Purification of Protein

The previously constructed recombinant plasmids were transformed into *E. coli* BL21(DE3) competent cells (TransGen) by heat shock at 42°C for 30 s and screened with kanamycin (50 μg/ml). Single colonies were selected from the plate and inoculated into LB medium with kanamycin (50 μg/ml) at 37°C. When the OD600 was 0.6, isopropyl-b-D-thiogalactoside (0.2 mM) was added to the culture medium and further incubated for 6 h at 37°C. The cells were centrifuged for 20 min at 8,000 ×*g* to collect the bacteria. Sonication was performed, and the supernatant was separated and precipitated by centrifugation for 20 min at 8,000 ×*g*. The precipitate was resuspended in Tris-HCl (20 mmol/l, pH 8.0) buffer containing 8 M urea at a ratio of 1:10 (w/v). The mixture was incubated at 4°C overnight and then filtered through a 0.45 μm membrane. The denatured p170 and p170-RBD proteins were then further purified with Ni-affinity chromatography (GE Healthcare, USA) using the ÄKTA explore system (GE Healthcare). The column was equilibrated with Tris-HCl buffer (20 mmol/l, pH 8.0) containing 8 M urea. The concentration of imidazole was increased gradually, and the corresponding peak was collected. Purified protein was refolded by reducing the urea concentration gradually with a dialysis bag, followed by concentration with 10 kDa and 100 kDa ultrafiltration devices successively and then stored at -80°C.

### Western Blotting

Previously prepared p170 and p170-RBD proteins were analyzed by SDS–PAGE and transferred to NC membranes. Non-reducing gel electrophoresis was performed by adding loading buffer with 0.1% SDS and no β-mercaptoethanol to the sample with no heating, while the reducing SDS–PAGE was performed by adding loading buffer containing 0.1% SDS and β-mercaptoethanol and heated. The membranes were subsequently blocked in PBS (containing 10% skimmed milk) for 1 h at 37°C and washed 3 times with PBST (PBS containing 0.5% Tween 20) solution for 5 min each time. The NC membranes were then placed in a 1:10000 dilution of anti-His mouse monoclonal antibody solution with gentle mixing while incubating at 37°C for 1 h, washed 3 times, incubated in a 1:10000 dilution of HRP-labeled goat anti-mouse IgG antibody solution at 37°C for 1 h, and then washed 5 more times. The signal was then immediately developed with an electrochemiluminescence (ECL) reagent and photographed on a Gelcap System (Tanon, China).

### Human Antibody Binding ELISA

To assess whether p170-RBD could bind to the anti-RBD antibodies in the sera of donors immunized with the commercial inactivated vaccine, 100 μl purified p170-RBD (10 μg/ml) or p170 (10 μg/ml) diluted in carbonate buffer was coated on plates at 4°C overnight. The plates were then blocked with 300 μl of 10% skimmed milk in PBS at 4°C for 16 h. Heat-inactivated human sera (starting dilution 1:10) were diluted in PBS (containing 0.1%BSA) in a 2-fold serial dilution. Then, 100 μl of the diluted serum samples was added to the plates for 45 min at 37°C. The plates were then washed with PBST three times, and 50 μl of HRP-conjugated mouse anti-human antibody (Solarbio, China) diluted 1:10000 in PBS (containing 0.1% BSA) was added to the wells for 30 min at 37°C. Plates were washed and developed as described previously.

### HEV Antibody Binding ELISA (Mouse Sera)

To assess whether p170-RBD and p170 could bind anti-HEV antibodies, sera from mice immunized with the recombinant vaccine (Changchun Institute of Biological Products, China) were collected, and an ELISA was performed. The procedure used was as described previously. The cut-off value was calculated by the following equation:

Cut-off = *n* * 2.1

where n is the OD450 of the negative control wells, with the titer set as the dilution of the wells greater than the cut-off value.

### ACE2 Binding Assay

Immunofluorescence staining was performed to verify the binding ability of p170-RBD to ACE2. 293T cells overexpressing ACE2 were cultured until they were approximately 70% confluent in 24-well plates. The cells were washed with PBS once and fixed with 500 μl/well 4% paraformaldehyde for 20 min. The paraformaldehyde was discarded, and the cells were washed with PBST 3 times. The cells were blocked with 500 μl/well PBS (containing 10% skimmed milk), placed at room temperature for 20 min, and washed another 3 times. The p170-RBD and p170 protein were diluted to 10 μg/ml with PBS, and 500 μl/well was added, respectively, after which the plates were stored at 37°C for 90 min, and then washed with PBST 3 times. An anti-p170 mouse polyclonal antibody (diluted in PBS, 1:1000, Changchun Institute of Biological Products) was added as a primary antibody, followed by incubation at 37°C for 1 h and 3 washes. Then, anti-ACE2 rabbit polyclonal antibody (diluted in PBS, 1:100, Abclonal, China) was added for 1 h and washed 3 times. A FITC-labeled goat anti-mouse antibody (diluted in PBS, 1:100) and Cy3-labeled goat anti-rabbit antibody (diluted in PBS, 1:100) were added as secondary antibody, followed by incubation at room temperature for 1 h and 3 washes. DAPI (200 μl/well, diluted in PBS, 1:1000) was added and incubated for 5 min, followed by one wash, after which the sample was immediately observed under a confocal laser scanning microscope.

### Transmission Electron Microscope (TEM)

A TEM was used to observe the structure of the protein. Briefly, 10 μl of sample (0.5 μg/μl) was applied to a copper screen and stained with 1% phosphotungstic acid, followed by imaging with a NANOSPRT5 camera.

### Dynamic Light Scattering (DLS)

DLS was performed to analyze the diameter distribution of the VLPs. One milliliter of sample (0.5 μg/μl) was added to a cuvette and analyzed by a Mastersizer 2000.

### Vaccine Immunogenicity and Sampling

Aluminum hydroxide (1 mg/ml) and p170-RBD or p170 (0.02 mg/ml) were mixed at a ratio of 1:1 (v/v). Female KM mice were divided into 3 groups (*n* = 6) and intraperitoneally immunized with the candidate vaccine at a dose of 100 μl, and negative control groups were immunized with 100 μl aluminum hydroxide adjuvant (0.5 mg/ml). Blood samples were collected every week after the prime vaccination.

### Humoral Immunity of p170-RBD (Mouse Serum)

To assess IgG antibody in serum samples, RBD antigen (Origene, China) was diluted to 3 μg/ml in carbonate buffer, and 100 μl was coated in plates and incubated at 4°C overnight, followed by blocking with PBS (containing 10% skimmed milk) at 4°C for 16 h and storage at -80°C. The serum was diluted serially with PBS (containing 0.1%BSA), and 100 μl was added to the plate. The cells were incubated at 37°C for 1 h and washed 3 times, after which HRP-labeled goat anti-mouse IgG antibody (diluted with PBS containing 0.1% BSA, 1:8000) was added and incubated at 37°C for 30 min, followed by 5 washes. The plates were washed and developed as described previously.

### Pseudovirus Neutralization Test (pVNT)

To perform this assay, 293T cells overexpressing ACE2 were cultured in 96-well plates. Then, mouse sera were diluted 10^3^ times in OptiMEM, 50 μl was added to a 96-well plate, and 10 μl/well pseudovirus (Sinobio, China) was added to the sera and incubated at 37°C for 1 h. The mixture was then transferred to the target cells and incubated at 37°C for 48 h. Firefly luciferase activity was measured using a detection reagent (Promega, USA) on the VICTOR X2 System (Perkin Elmer, USA). The inhibition efficiency of the serum antibodies against pseudotyped viruses was analyzed using GraphPad Prism 8.0 software.

### Statistical Analysis

Titers were analyzed by one-way ANOVA with a Student-Newman–Keuls multiple comparison test. A 3-parameter logistical ﬁt of the data was performed as previously described for the ELISAs and the neutralizing antibody test. Statistical analyses were performed with GraphPad Prism 8.0 software, the mean ± standard deviation (x ±SD) was calculated, and statistical significance was determined (*p* < 0.001).

## Results

### p170 and p170-RBD Could Be Efficiently Expressed in Bacteria

**Fig. 1:**
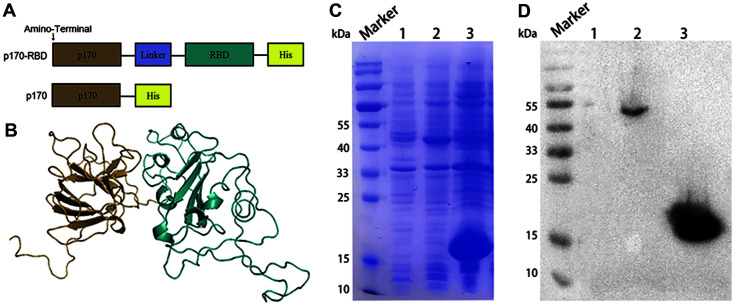
Structure simulation and expression of p170 and p170-RBD. (**A**) Schematic of the vaccine components. (**B**) Structure simulation of p170-RBD. The brown portion represents the p170 protein structure, and the blue portion represents the RBD structure. The separation of p170 and RBD proved that the addition of the linker allowed for the structural integrity of the two proteins. (**C**) The expression of p170 and p170-RBD in bacterial lysates. Lane 1, control group; Lane 2, p170-RBD, approximately 44 kDa; Lane 3, p170, approximately 19 kDa. (**D**) Western blot of p170 and p170-RBD from bacterial lysates with an anti-His monoclonal antibody. Lane 1, control group; Lane 2, p170-RBD; Lane 3, p170. Both p170 and p170-RBD had a strong reactivity with the anti-His monoclonal antibody.

p170-RBD is a fusion protein composed of p170 and RBD that is connected by a linker (GGGGS). A His-Tag (6 × H) was added at the carboxyl-terminus of p170 or p170-RBD (Fig. 1A). Likely due to the presence of the linker, as seen in structural simulation (Fig. 1B), the brown portion that represents the p170 protein was separated from the blue portion representing the RBD, implying that the two proteins were fused and expressed segregated. A total of 722 residues (95%) were modeled at > 90% accuracy, indicating that the result was credible. The *E. coli* BL21(DE3) strain was used to express p170-RBD. SDS–PAGE showed that the two proteins could be expressed successfully. The yield of p170-RBD and p170 was 40.9% and 9.1%, respectively. p170 presented as a monomer, with an approximate molecular weight of 19 kDa (Fig. 1C, Lane 2), while p170-RBD had a molecular weight of approximately 44 kDa (Fig. 1B, Lane 3). Western blotting confirmed that the expressed proteins had good reactivity with the anti-His mAb (Fig. 1D, Lanes 2, 3).

### p170-RBD Could Be Purified and Form a Dimer Structure

**Fig. 2:**
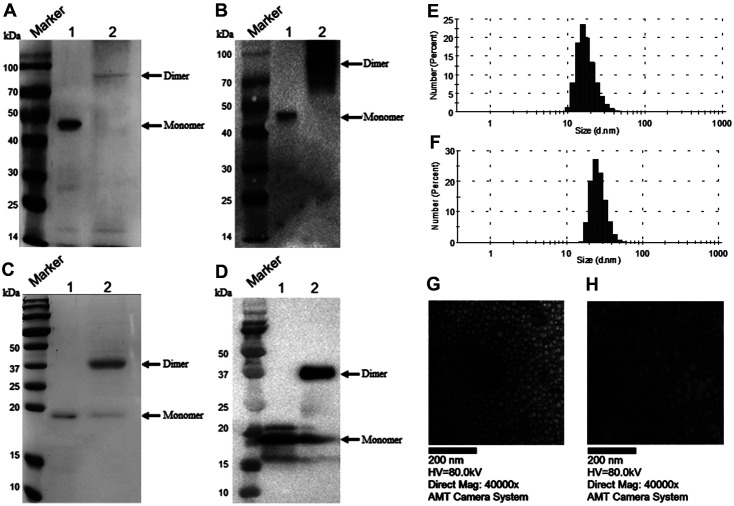
p170 and p170-RBD could form a dimer structure and assembled into VLPs. (**A**) and (**C**) SDS–PAGE of p170 and p170-RBD, respectively. Lane 1, reducing SDS–PAGE for p170 and p170-RBD; Lane 2, non-reducing SDS–PAGE for p170 and p170-RBD. The dimer in non-reducing SDS–PAGE was twice the size of the monomer in reducing SDS–PAGE. (**B**) and (**D**) Western blot of p170 and p170-RBD, respectively, with an anti-His monoclonal antibody. Lane 1, reducing SDS–PAGE for p170 and p170-RBD; Lane 2, non-reducing SDS–PAGE for p170 and p170-RBD. (**E**) and (**F**) Nanoparticle size analysis of p170 and p170-RBD, respectively. The diameter of the p170 particles was approximately 18 nm and that of p170-RBD was approximately 24 nm. (**G**) and (**H**) TEM analysis of p170 and p170-RBD, respectively.

In our previous study, the RBD protein alone could not be observed in the supernatant from a prokaryotic expression system (data not shown). The inclusion body protein may not be the best candidate for a vaccine, but feasible strategies for renaturation could help p170-RBD form VLPs. In this study, p170 was used as the signal peptide to allow p170-RBD to form a dimer structure after renaturation. After collecting the 150 mm imidazole elution peak, SDS–PAGE revealed that the purities of p170-RBD and p170 were high. p170-RBD, as analyzed by non-reducing gel electrophoresis, was observed as a protein of approximately 88 kDa (Fig. 2A, Lane 2), which was twice the size of p170-RBD (Fig. 2A, Lane 1), indicating that p170-RBD could assemble into a dimer. p170 showed the same trend as p170-RBD, with a monomer of 19 kDa (Fig. 2C, Lane 1) and a dimer of 38 kDa (Fig. 2C, Lane 2). Western blot analysis showed that both monomers and dimers of p170-RBD and p170 could be recognized by the anti-His mAb (Figs. 2B and 2D).

### Characterization of the p170-RBD Nanoparticles

The proteins were also analyzed by a nanoparticle size analyzer. We observed that particles formed by p170 had a diameter range of 10 nm to 40 nm, with most particles having a diameter range of 18 nm (Fig. 2E). For p170-RBD, the diameter ranged from 16 nm to 50 nm, with most particles having a diameter of 24 nm (Fig. 2F). To further observe the morphology of the particles, a TEM analysis was performed, and we could see that the size of the particles as judged by electron microscopy was consistent with that determined by DLS (Figs. 2G, 2H), although the shape of the particles was irregular, which may cause the size determined by DLS to show a nonhomogeneous distribution.

### Temperature Stability of p170-RBD

**Fig. 3:**
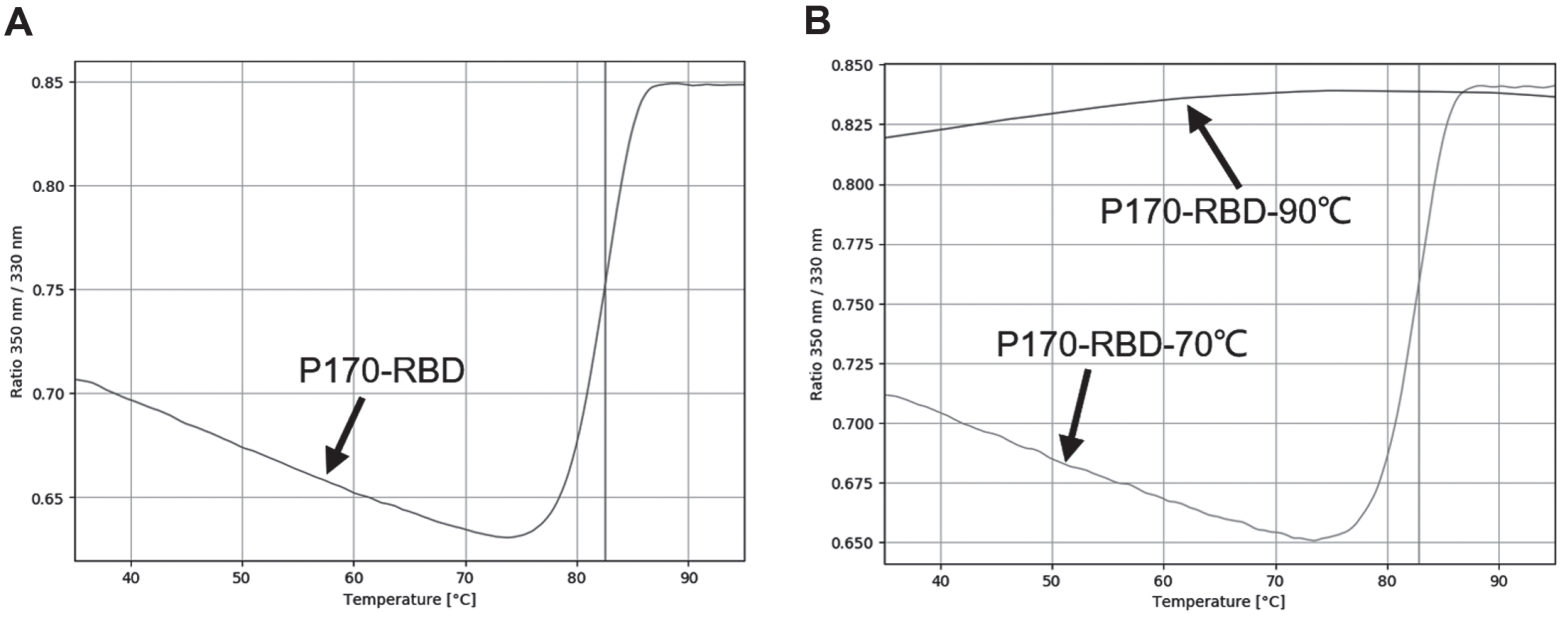
Thermal stability of p170-RBD. (**A**) was p170-RBD heated from 30°C to 100°C, the ratio of 350nm to 330nm showed significant change, implying he helical structure of p170-RBD was destroyed from 75°C, and completely denatured at about 87°C, with an inflection temperature of 82.5°C. (**B**) was p170-RBD heated at 70°C and 90°C for 1min respectively. Results showed that heating at 70°C did not impact the inflection temperature while heating at 90°C for 1 min could destroy the structure of p170-RBD, implying that the results of protein stability detection are reliable.

To verify the temperature stability of p170-RBD, NanoTemper was used. It was found that p170-RBD denatured at 75°C and finally completely denatured at approximately 87°C (Fig. 3A), with an inflection temperature of 82.5°C, showing that p170-RBD possessed good thermal stability. Then, we heated it at 90°C for 1 min and at 70°C for 1 min for accuracy verification and the thermal stability was analyzed again, which showed that the structure of p170-RBD was damaged at 90°C but not at 70°C (Fig. 3B), proving the feasibility of this method.

### Antigenicity Assay of p170-RBD

**Fig. 4:**
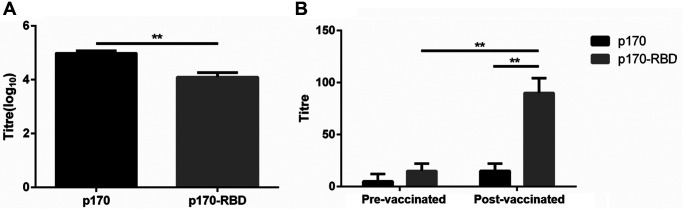
Antigenicity of p170 and p170-RBD as determined by ELISA. (**A**) ELISA for the reactivity of p170 and p170- RBD with anti-HEV pAbs. (**B**) ELISA for the reactivity of p170 and p170-RBD with neutralizing antibodies. The left panel shows serum from pre-vaccinated people; the right panel shows serum from post-vaccinated people (** *p* < 0.001).

To confirm the antigenicity of p170-RBD, we performed two binding assays. p170-RBD was tested using anti-HEV polyclonal antibodies (pAbs) from mice. Compared with the titer of 5 log_10_ for p170, p170-RBD only reached a titer of approximately 4.12 log_10_ (*p* < 0.001), and we presumed that the p170 epitope in p170-RBD was masked (Fig. 4A). Then, we evaluated the binding ability of p170-RBD to human sera antibodies. The results showed that p170-RBD could bind to pAbs strongly at a titer of 1:80 (Fig. 4B), while a titer of only 1:15 was observed for p170, which was consistent with the control group, indicating that p170-RBD could bind to the serum antibodies.

### p170-RBD Protein Could Bind to ACE2

**Fig. 5:**
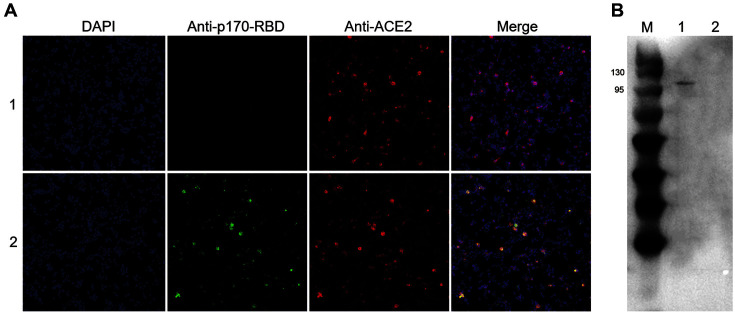
Identification of ACE2 binding ability of p170 and p170-RBD by immunofluorescence staining. (**A**) Immunofluorescence staining result, (1) shows p170-RBD, while (2) shows the control group with p170. (**B**) Western blot of 293T cells transfected with pcDNA3.1-ACE2 or pcDNA3.1, respectively, with an anti-ACE2 polyclonal antibody. Lane 1, 293T cells transfected with pcDNA3.1-ACE2; Lane 2, 293T cells transfected with pcDNA3.1.

Although we verified the binding of p170-RBD to antibodies from humans and mice, antigenicity was not the main factor that was used for evaluating production of neutralizing antibodies, and thus we evaluated whether p170-RBD could bind to ACE2 by immunofluorescence staining. Compared with the control group (Fig. 5A, Line 2), the addition of p170-RBD contributed to the fluorescence that appeared on the cell surface (Fig. 5A, Line 1). We also found that green fluorescence appeared along with red fluorescence, meaning that ACE2 co-localized with p170-RBD, suggesting that p170-RBD could bind to ACE2, which led to the binding of fluorescent antibodies. Although the efficiency of the connection could not be measured quantitatively, we confirmed that p170-RBD could bind to ACE2 at the highest content of 0.01 mg/ml. To identify whether ACE2 expressed as a truncated form, 293T cells transfected with pcDNA3.1-ACE2 or pcDNA3.1 were cultured, collected and lysed, and then western blotting was performed. As seen in Fig. 5B, 293T cells transfected with pcDNA3.1-ACE2 in lane 1 could form a bond at approximately 110 kDa, while 293T cells transfected with pcDNA3.1 didn’t exhibit this bond, indicating that ACE2 was expressed in cells successfully.

### p170-RBD Protein Could Stimulate the Body to Produce Humoral Immunity and Nneutralizing Antibodies

**Fig. 6:**
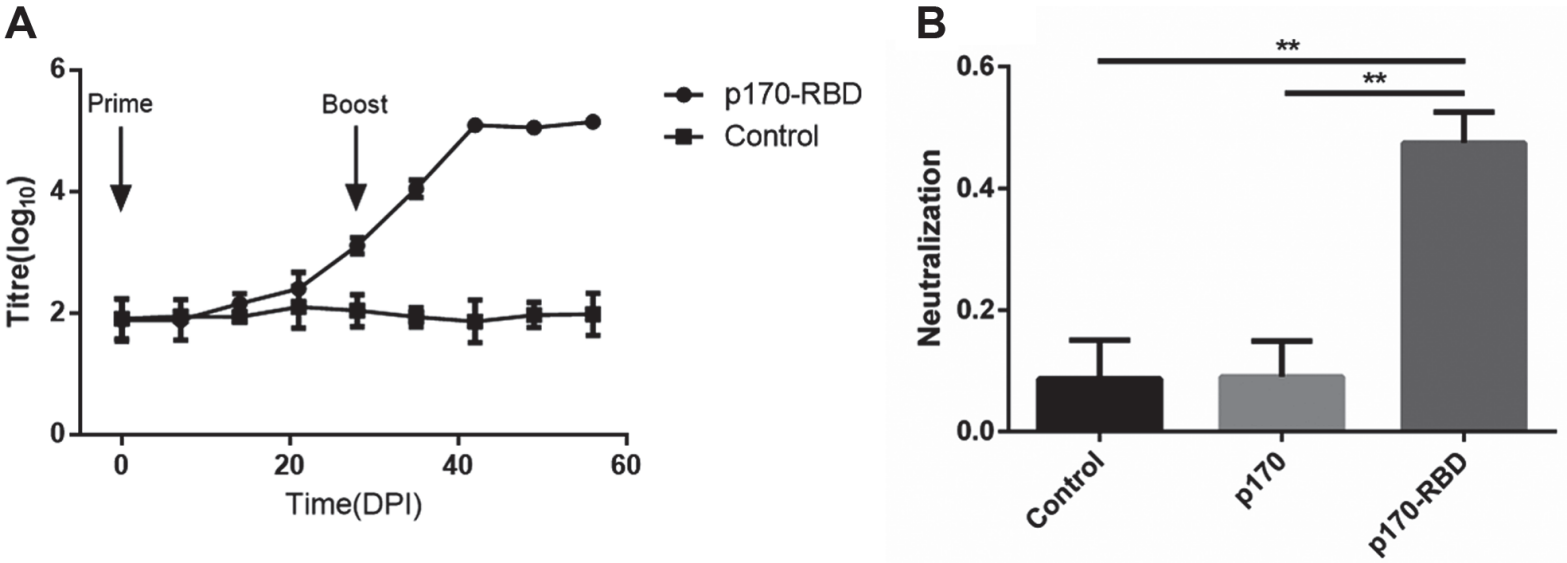
Immunogenicity assay of p170 and p170-RBD in mice. (**A**) The titer of anti-RBD antibodies varies with time. The immunization time points are indicated by arrows. p170-RBD induced a titer as high as 4.97 log_10_ at 42 dpi, which lasted to at least 56 dpi, whereas the naive group showed no obvious variation. (**B**) Neutralization activity of p170-RBD (** *p* < 0.001).

Finally, we evaluated the immunogenicity of p170-RBD. We observed that the IgG antibody titer varied with time: the titer increased gradually from 14 days post-immunization (dpi) after the prime vaccination, increasing dramatically after the boost vaccination, with the peak titer of 4.97 log_10_ achieved at 42 dpi, which lasted at least 2 weeks, while p170 reached a titer of only 1:100 at 42 dpi (Fig. 6A). Then, we evaluated the inhibition rate of the neutralizing antibodies, and the results showed that when the serum was diluted 103 times, approximately 47.5%of the pseudovirus could be neutralized, which was much higher than the 8.6% neutralization observed with the neutralizing antibodies induced by p170 (*p* < 0.001), implying that the vaccine could stimulate the body to produce neutralizing antibodies (Fig. 6B).

## Discussion

Although several vaccines against SARS-CoV-2 have been used widely, the global supply of vaccines is still short [27]. Currently, all recombinant vaccines targeting 2019-NCoV have been designed using its RBD region [28], but in our previous study, we found that the SARS-CoV-2 RBD regions alone could not form VLPs. In this study, the ORF2 region of HEV3 (aa 439-608) was fused with the RBD for expression, with the aim to form VLPs, and the results showed that the fusion protein could indeed form a dimer after renaturation and assemble into VLPs with a diameter of approximately 24 nm. The particles were not sufficiently round, as observed in the TEM analysis, which may be related to the renaturation process. We plan to explore the process of renaturation in a future study.

To measure the binding activity of p170-RBD, we performed a series of ELISAs. The binding assay with the serum of donors immunized with inactivated vaccines showed that p170-RBD could bind to the neutralizing antibodies, indicating that p170-RBD contained cross-epitopes for SARS-CoV-2. For the HEV antibodies, we found that titer induced by p170-RBD was significantly lower than that induced by p170 and suspect this might be due to the structure of the VLP. Finally, an indirect immunofluorescence assay was performed, which indicated indirectly that p170-RBD could bind to ACE2, even though this method could not quantitatively determine the binding force.

As one of the most commonly used adjuvants of human vaccines, aluminum hydroxide can stimulate the body to produce humoral and cellular immunity [29]. The immunization procedure of the candidate vaccine used a two-shot method of immunization at 0 and 4 weeks. After the second immunization, the antibody titer in mice increased significantly, reaching 4.97 log_10_ at 42 dpi and lasting for 2 weeks, indicating that the vaccine could stimulate the body to produce strong humoral immunity. Neutralizing antibodies have been used widely to evaluate the immune effect of vaccines. Nie *et al*. established and performed a pseudovirus neutralization assay for SARS-CoV-2, and a 50% tissue culture infectious dose (TCID50) of SARS-CoV-2 pseudovirus was determined. A viral inoculation of 650 TCID50/well was determined as the optimal dose [30]. In this study, pVNT was also used, and the results showed that neutralizing antibody in the serum at 42 dpi could inhibit approximately 47.5% of the virus infecting the cells when the serum was diluted 10^3^ times. Here, the appropriate concentration of p170-RBD was not explored, although this should be examined for further improvement of this fusion protein as a vaccine candidate. We did not test the protective effects of the vaccine in a mouse or hamster challenge model, which would provide compelling evidence regarding the immunogenicity of the vaccine.

In summary, we successfully expressed p170-RBD in *E. coli* and prepared the p170-RBD protein by fermentation and purification. p170-RBD could assemble into VLPs after renaturation. p170-RBD could bind to human serum and ACE2, implying that its structure was not only the same as that of SARS-CoV-2 but that it also had good antigenicity. In immunogenicity assays, the candidate vaccine possessed strong immunogenicity for humoral immunity and could suppress the infection of cells by a pseudovirus.

## Figures and Tables

**Table 1:** Primers used in research.

Name	Primers (5' to 3')
p170-RBD-F	GCGAATTCATGGTTATTCAGGATTACGATA
p170-RBD-R	GCCTCGAGTTAGCTCTTTTTCGGGCC
p170-F	GCGAATTCATGGTTATTCAGGATTACGATA
p170-R	GCCTCGAGTTAAAGAGCCGAGTGTGGGG
ACE2-F	GCGGTACCGCCACCATGTCAAGCTCTTCCTGGC
ACE2-R	GCCTCGAGCTAAAAGGAGGTCTGAACATCA

The italic is the site of enzyme.
